# Meta-analysis of the therapeutic effect of electrical stimulation combined with pelvic floor muscle exercise on female pelvic floor dysfunction

**DOI:** 10.1186/s40001-024-01979-1

**Published:** 2024-07-22

**Authors:** Yaqin Huang, Zhoulu Huang, Yi Ou, Lin Yin, Yuxiao Sun, Huiyan Zong

**Affiliations:** 1 West China Hospital, Sichuan University, Chengdu, 610000 China; 2 Department of Rehabilitation Medicine, West China Hospital, Sichuan University, No. 37, Guo Xue Alley, Chengdu, 610041 Sichuan China

**Keywords:** Pelvic floor dysfunction, Electrical stimulation, Pelvic floor muscle exercise, Meta-analysis

## Abstract

**Objectives:**

To systematically evaluate the therapeutic effect of electrical stimulation combined with pelvic floor muscle exercise on female pelvic floor dysfunction (PFD).

**Methods:**

Preferred Reporting Items for Systematic Reviews and Meta-Analyses (PRISMA) was applied. A computer-based retrieval was performed in the databases of PubMed, Web of Science, Embase, and Cochrane Library from database establishment to September 15, 2023, to identify randomized controlled trials on electrical stimulation combined with pelvic floor muscle function exercise on female PFD. Literature screening, data extraction, and quality evaluation were performed independently by two researchers, and meta-analysis was performed using the statistical software Stata15.0.

**Results:**

1. In total, 12 randomized controlled trials were included, involving 721 female patients. The overall quality of methodologies employed in the included studies was relatively high. 2. Meta-analysis results showed that electrical stimulation combined with pelvic floor muscle exercise could effectively mitigate the severity of female PFD (SMD = -1.01, 95% CI *− *1.78, − 0.25, *P* < 0.05). 3. This combination treatment demonstrated a significant positive effect on the improvement of pelvic floor muscle strength in female patients (*P* < 0.05); however, it had no significant effect on the improvement in quality of life (*P* > 0.05).

**Conclusions:**

Compared with pelvic floor muscle exercise alone, electrical stimulation combined with pelvic floor muscle exercise could effectively mitigate the severity of female PFD. It had a notable positive impact on enhancing pelvic floor muscle strength in female patients, although it did not significantly improve quality of life. Future high-quality studies are warranted.

**Supplementary Information:**

The online version contains supplementary material available at 10.1186/s40001-024-01979-1.

## Introduction

Pelvic floor dysfunction (PFD) is a condition characterized by reduced support capacity and structural changes in pelvic floor muscles [1]. The related diseases mainly include but are not limited to urinary incontinence, pelvic organ prolapse, sexual dysfunction, and pelvic pain [2]. PFD is more prevalent in adult women, with a reported prevalence rate of up to 60.2%, which imposes significant life burdens and psychological pressures on affected individuals, presenting a substantial challenge to public health [3, 4]. Therefore, how to improve female PFD has recently become a research hotspot.

Pelvic floor muscle exercise (PFME) is recognized as the primary treatment choice for PFD [5], but existing studies indicate that most female PFD patients have poor treatment compliance with PFME [6]. Electrical stimulation (ES) promotes active muscle contraction and enhances muscle strength primarily by delivering an electrical current and stimulating pelvic floor muscles [7]. As a treatment option with advantages such as low trauma and easy operation, it mainly acts on the patient's internal or external vagina (sacral nerve, tibial nerve, perineum, buttocks, and thighs) [8, 9]. At present, many studies in China and overseas have investigated the efficacy of PFME combined with ES on female PFD, but the results are still controversial.

Based on this, this study is designed to systematically analyze the modulation of different intensities of interventions on female PFD through the comparison of the efficacy of PFME combined with ES on female PFD using meta-analysis, providing a scientifically valid theoretical basis for formulating intervention plans for patients with female PFD.

## Methods

### Study registration

This meta-analysis was performed according to the PRISMA guidelines [10], and a protocol was registered in the PROSPERO database (CRD42024501351).

### Inclusion and exclusion criteria

#### Subjects

Female patients with a diagnosis of PFD were included in the study, irrespective of race, nationality, or course of the disease.

#### Interventions

Treatment group: patients treated with any form of PFME and ES therapy; control group: patients treated with PFME.

#### Outcome measures

In this study, the primary outcome measure was the severity of PFD. The secondary outcome measures included pelvic floor muscle strength and quality of life. Studies with incomplete data or that could not be analyzed were excluded.

#### Study types

Only randomized controlled trials (RCTs) were included in the study.

#### Exclusion criteria

Non-English studies, duplicate published studies, and studies with incomplete original data that cannot be utilized.

### Data source and retrieval strategy

The computer-based retrieval of databases of PubMed, Web of Science, Embase, and Cochrane Library was conducted up to September 15, 2023, without regional or racial restrictions. The subject headings and free words were jointly adopted for the retrieval. Search terms included: "Electric Stimulation", "Electric Stimulation Therapy", "Electrostimulation", "Exercise Therapy", "pelvic floor exercise", "pelvic floor training", "Pelvic Floor Disorders", "Pelvic Girdle Pain", "Pelvic Organ Prolapse", "Pelvic Pain", "Urinary Incontinence", "Fecal Incontinence", "Uterine Prolapse", "Cystocele” and "Sexual Dysfunction, Physiological", as presented them in Table S1. During the literature screening, the results of different electronic databases were entered into EndNote Version X9 to remove duplicated studies. After the removal of duplicates, the retrieved studies were reviewed by two researchers independently based on the predefined inclusion and exclusion criteria.

### Data extraction

Data from the included studies were extracted and summarized by two researchers independently using a standardized data extraction form. Extracted study information included the first author, publication year, country, average age (treatment/control group), sample size (treatment/control group), interventions (treatment/control group), ES parameters in the treatment group, and outcome measures.

### Statistical analysis

Statistical analysis was performed with Stata 15.0. Continuous variable data were analyzed using mean difference (MD) and standardized mean difference (SMD) with 95% confidence intervals (CIs), depending on whether it was measured with the same scale or different scales. Heterogeneity was tested with the *I*^2^ test. If *I*^2^ > 50, a random-effects model was used; otherwise, a fixed-effects model was used. A forest plot was drawn to evaluate the weight of each study and the summary results.

## Results

### Results of literature search

**Fig. 1:**
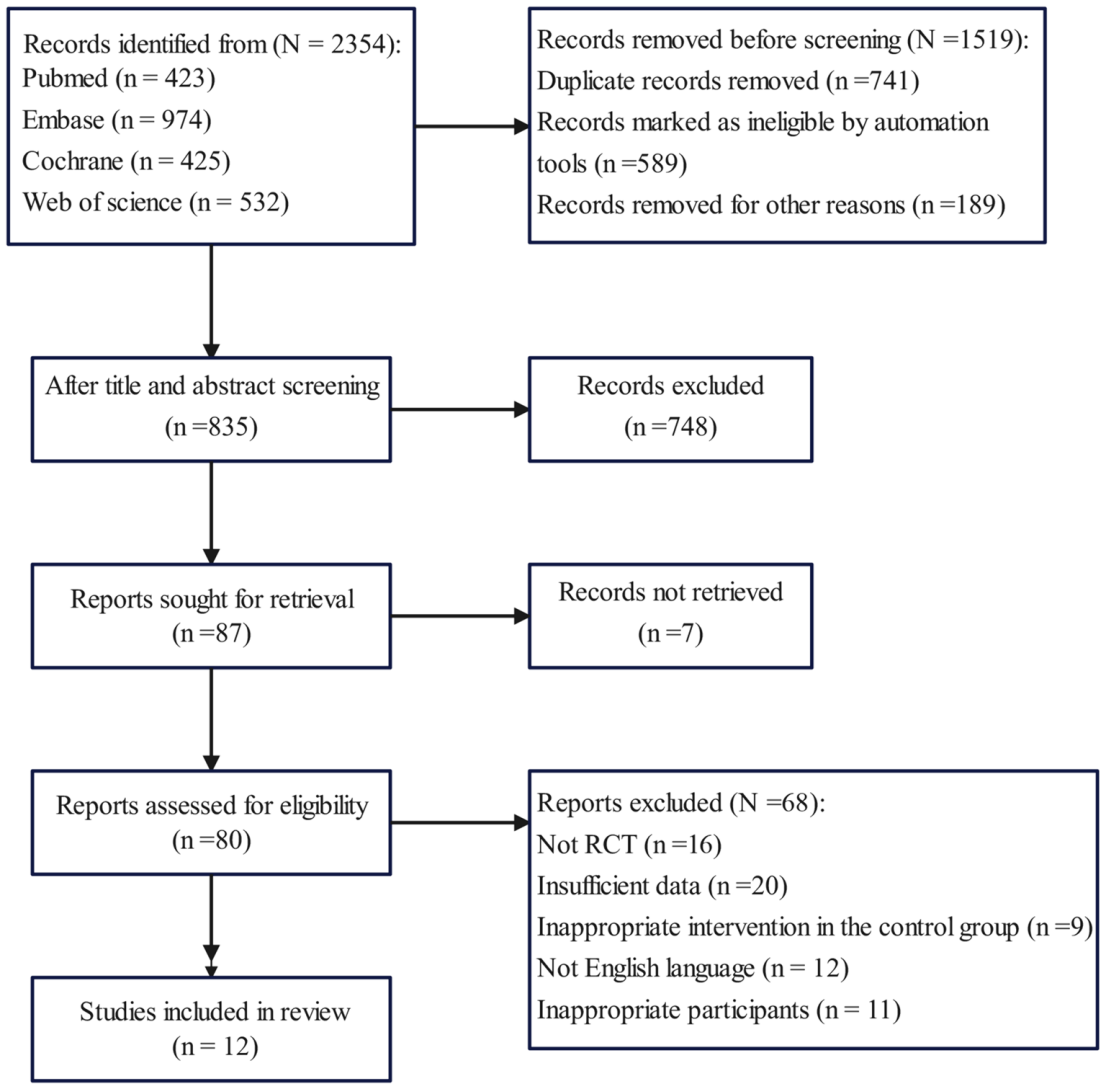
Process and results of literature screening

The PRISMA flowchart depicting the process and results of the literature search is shown in Fig. 1. In total, 2354 related studies were obtained. Among them, 1519 studies were excluded due to duplicate records (*n* = 741), records flagged as ineligible by the automated tool (*n* = 589), and other reasons (*n* = 189). The title and abstract were accessed for the remaining 835 studies, of which 748 were excluded, because they did not meet the inclusion criteria. The full text of 87 studies was accessed, of which seven were excluded because the full text was not available. Of the remaining 80 studies, 68 studies were excluded after the original text was read, due to type of study (*n* = 16), missing data (*n* = 20), inappropriate control group (*n* = 9), not written in English (*n* = 12), and inappropriate participants (*n* = 11). Finally, 12 studies were included.

### Basic characteristics of the included studies

In total, 12 RCTs [11–22] were included in the final analysis. Among them, two studies were conducted in Brazil [11, 15], one was in England [23], one was in Denmark [12], three were in Turkey [13, 16, 18], one was in Spain [14], two were in China [17, 19], one was in Egypt [24], and one was in India [20]. The intervention for the treatment group was ES combined with PFME, while the control group received PFME alone. The severity of PFD was reported in seven studies [11, 12, 14, 15, 17, 18, 21], using tools such as ICIQ–UI–SF, ICIQ, and UDI, pelvic floor muscle was reported in three studies [16, 20, 24] using PFMS, and quality of life was reported in six studies [12–14, 17, 18, 21], using tools such as KHQ, I-QOL, QOL, FIQL, and SCI–QoL. The basic characteristics of the included studies are provided in Table 1.

**Table 1:** Basic characteristics of the included studies

The first author/publication year	Country	Sample size (T/C, n)	Treatment group		Control group	Outcome measures
			Interventions	Parameters of electrical stimulation	Interventions	
Schreiner [11] 2010	Brazil	25/26	ES + PFME	10 Hz, if patient-tolerable; 30 min/session, 1 session/week	PFME	ICIQ–UI–SF
Swati [23] 2017	England	30/34	ES + PEME	–	PFME	PISQ-31
Elmelund [12] 2018	Denmark	14/13	ES + PFME	Intermittent stimulation − 40 Hz, 7.5–10 min/session; Continuous stimulation − 10 Hz, 10–20 min/session	PFME	ICIQ–UI–SF, SCI–QoL
Karaman [13] 2020	Turkey	20/28	ES + PFME	0.03 mA, if patient-tolerable; 30 min/session, 2 sessions/week, for 4 weeks	PFME	QOL
Mundet [14] 2020	Spain	39/36	ES + PFME	35 Hz, if patient-tolerable; 30 min/session, 5 sessions/week	PFME	ICIQ, FIQL
Schreiner [15] 2020	Brazil	51/50	ES + PFME	10 Hz, if patient-tolerable; 30 min/session, 1 session/week, for 12 weeks	PFME	ICIQ–UI–SF
Celenay [16] 2021	Turkey	22/22	ES + PFME	10 Hz, if patient-tolerable; 30 min/session, 3 sessions/week, for 6 weeks	PFME	PFMS
Zhu [17] 2022	China	55/55	ES + PFME	For type I muscle fibers, 8–32 Hz; for type II muscle fibers 20–80 Hz; if patient-tolerable; 10–20 min/session, 2 sessions/week, for 5 weeks	PFME	ICIQ–UI–SF, I-QOL
Elhosary [24] 2022	Egypt	20/20	ES + PFME	Intermittent low-frequency stimulation 15 Hz; intermittent high-frequency stimulation 40 Hz; 20 min/session, every other day, for 8 weeks	PFME	PFMS
Sahin [18] 2022	Turkey	17/17	ES + PFME	50 Hz, if patient-tolerable; 30 min/session, 3 sessions/week, for 8 weeks	PFME	PFMS, KHQ, ISI
Chen [19] 2023	China	40/40	ES + PFME	≤ 100 mA, if patient-tolerable; 30 min/session, 2 sessions/week, for 3 months	PFME	ICIQ–UI–SF, I-QOL
Bali [20] 2023	India	10/10	ES + PFME	50 Hz, if patient-tolerable; 20 min/session, 1 session/week, for 12 weeks	PFME	PFMS

T: treatment group; C: control group; ES: electrical stimulation therapy; PFME: pelvic floor muscle exercise

Outcome measures: 1. PFD Severity: Incontinence Questionnaire–urinary incontinence short form (ICIQ–UI–SF), International Consultation on Incontinence Questionnaire (ICIQ), Incontinence Severity Index (ISI); 2. Quality of life: Kings Health Questionnaire (KHQ), Incontinence Quality of Life Scale (I-QOL), Incontinence Quality of Life questionnaire (I-QOL), Fecal Incontinence Quality of Life (FIQL), Spinal Cord Injury Quality of Life (SCI–QoL); 3. Pelvic floor muscle strength: PFMS

### Quality assessment results of the included studies

**Fig. 2:**
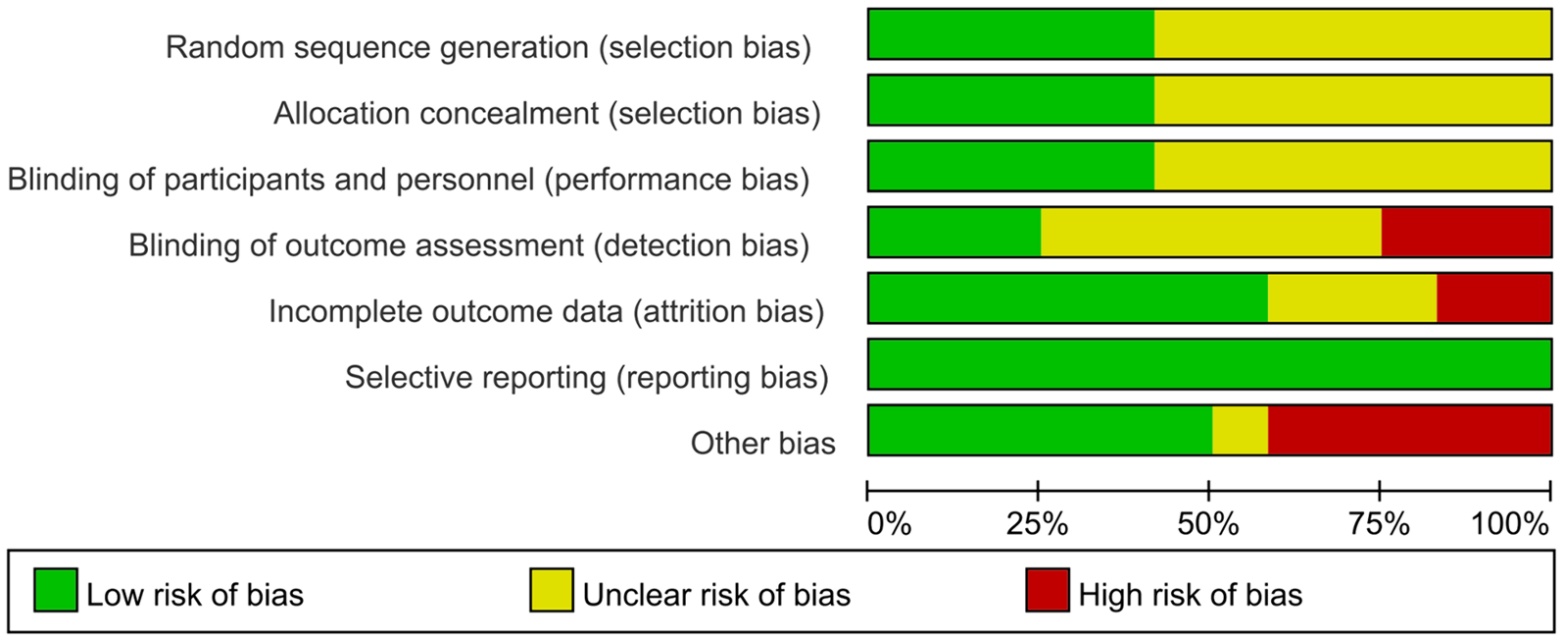
Bias in included studies

**Fig. 3:**
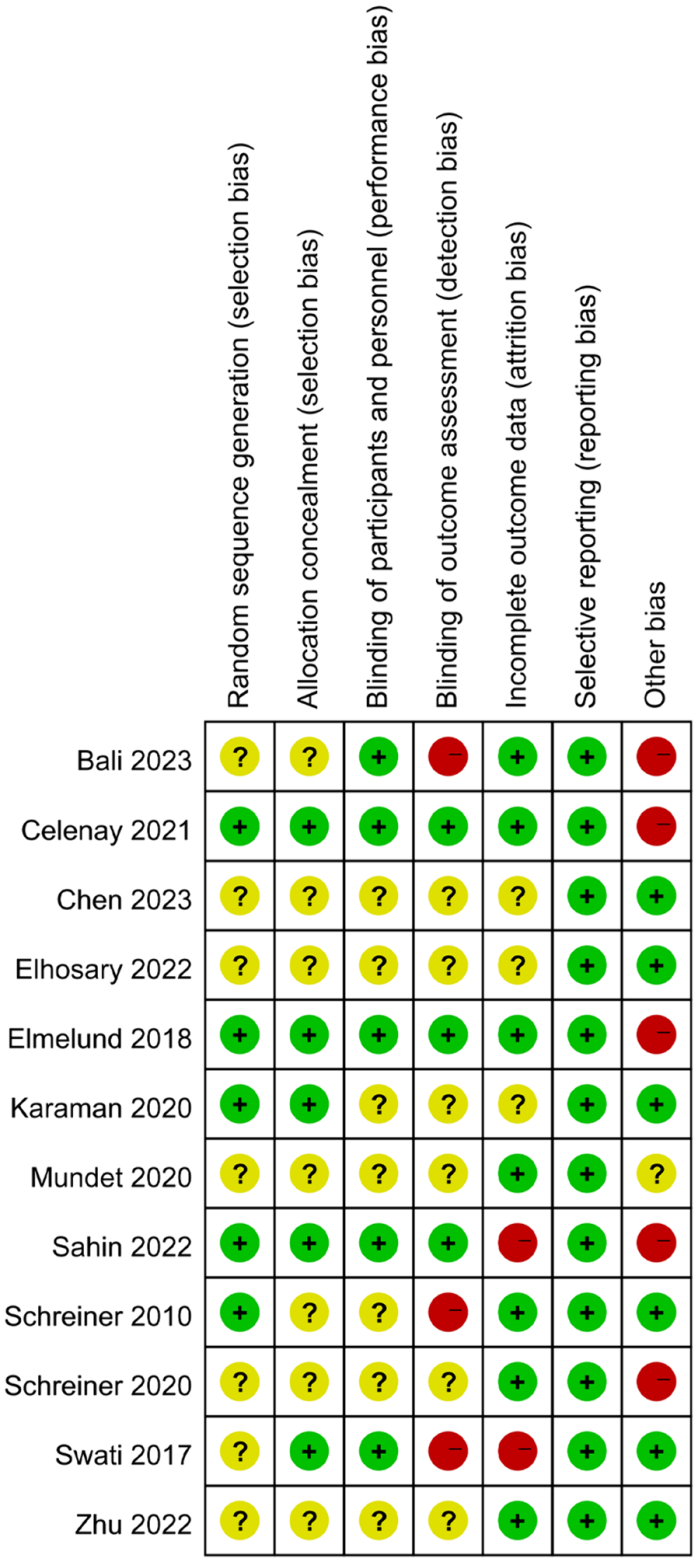
Bias risk assessment of included studies

In accordance with the risk assessment criteria of the Cochrane Collaboration, out of 12 RCTs, the random sequence generation was described in detail in five studies [11–13, 16, 18], which were assessed as low risk; an appropriate allocation concealment method was reported in three studies [16, 18] + 11, which were assessed as low risk; blinding to patients and primary investigators was described detailed in four studies [12, 16, 18, 20], which were assessed as low risk; the blinding to outcome assessments was not described clearly in two studies [20], which were assessed as high risk. The results are provided in Figs. 2 and 3.

### Severity of pelvic floor dysfunction

**Fig. 4:**
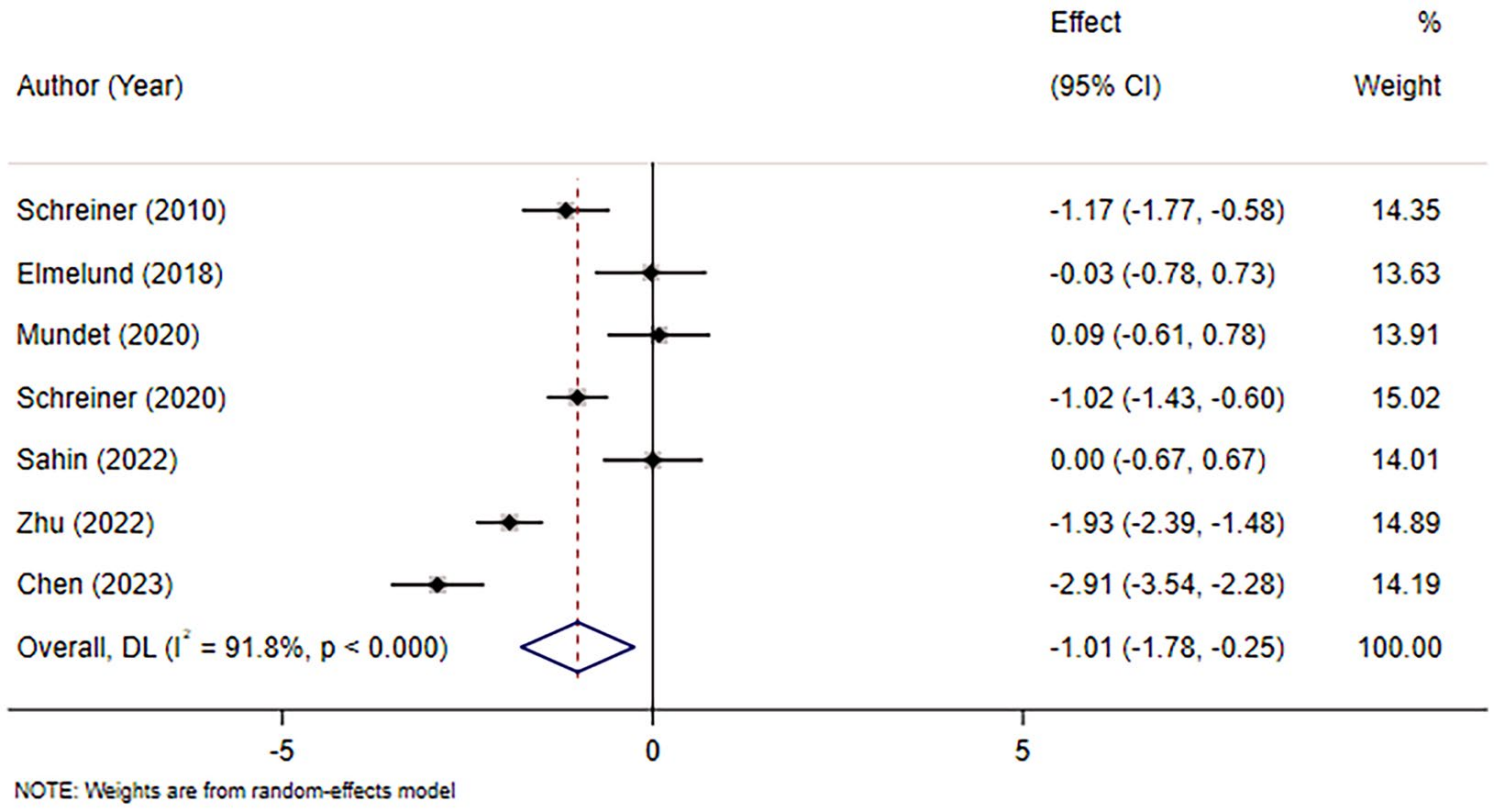
Meta-analysis of the effect of ES combined with PFME on the PFD severity in female patients

A total of seven studies [11, 12, 14, 15, 17–19] were included, involving 435 patients. Considering the significant heterogeneity between studies (*I*^2^ = 91.8%, *p* < 0.001), a random-effects model was applied. Because the units of the scales in the included studies were different, pooled statistics of *SMD* values were selected for analysis. The results revealed that there were statistically significant differences between the intervention and control groups in mitigating the severity of PFD in female patients (SMD = − 1.01, 95% CI (− 1.78, − 0.25), *p* < 0.05). The results are provided in Fig. 4.

### Pelvic floor muscle strength

**Fig. 5:**
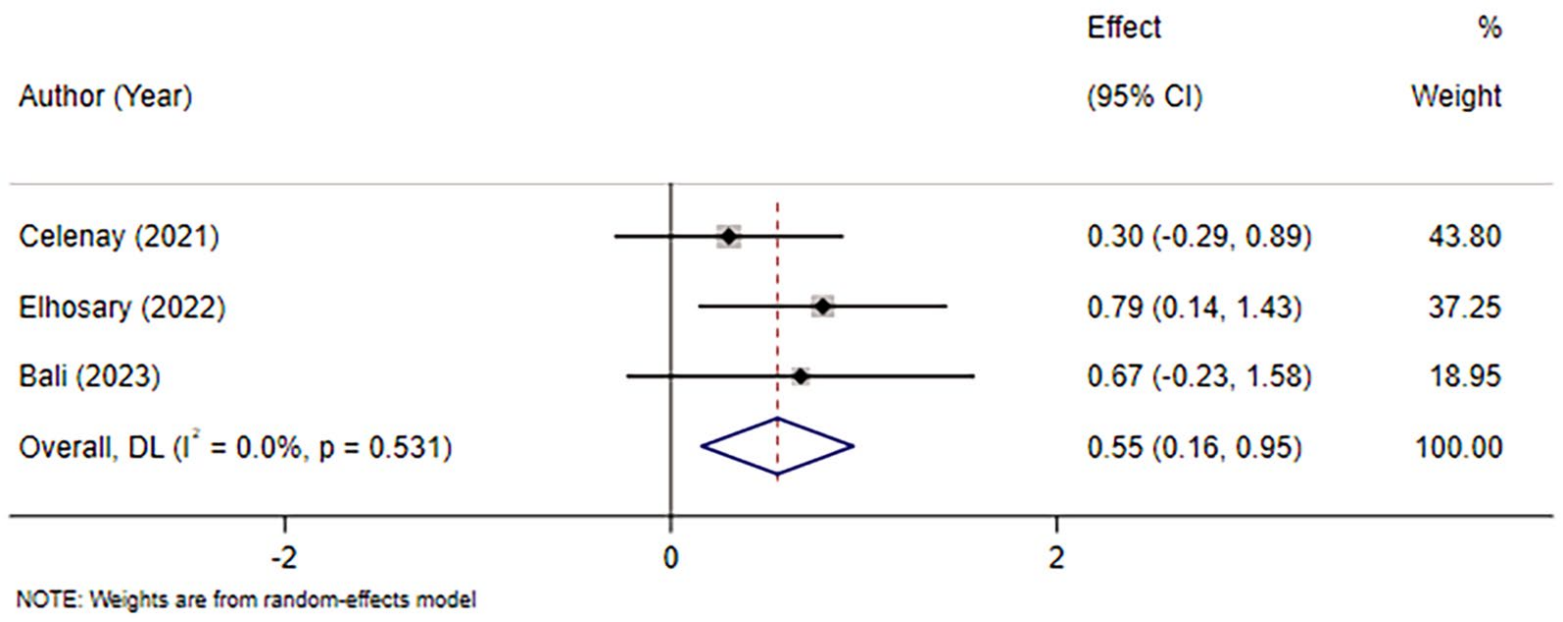
Meta-analysis of the effect of ES combined with PFME on pelvic floor muscle strength in women with PFD

Three studies [11, 16, 26] were included, involving 104 patients. Due to the low heterogeneity between studies (*I*^2^ = 0.0%, *p* > 0.05), a fixed-effects model was applied. The results revealed that there were statistically significant differences between the intervention and control groups in the effect on the pelvic floor muscle strength in women with PFD (MD = 0.55, 95% CI (0.16, 0.95), *p* < 0.05). The results are provided in Fig. 5.

### Quality of life

**Fig. 6:**
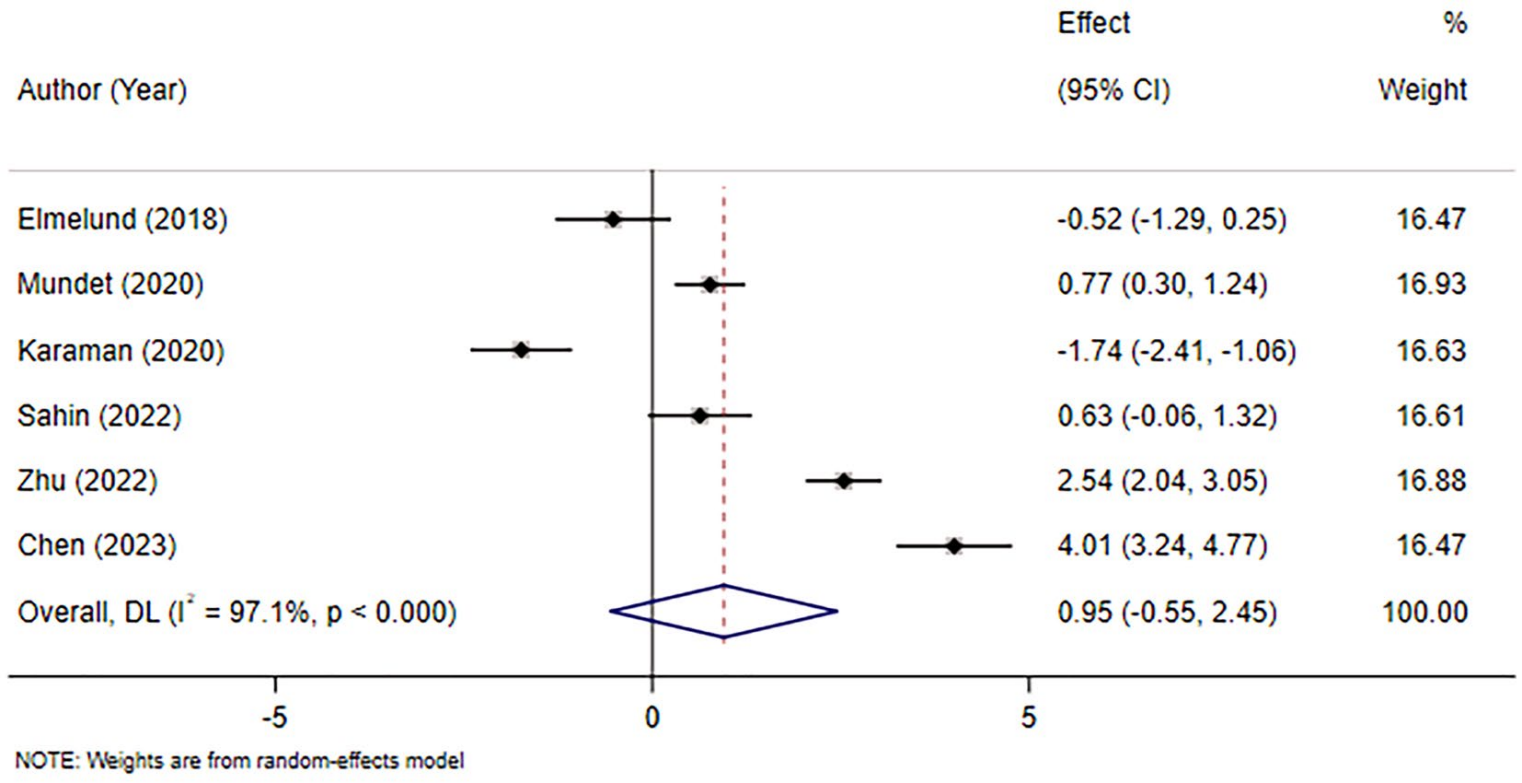
Meta-analysis of the effect of ES combined with PFME on the quality of life in women with PFD

Six studies [12–14, 17–19] were included, involving 374 patients. Considering the significant heterogeneity between studies (*I*^2^ = 97.1%, *p* < 0.001), a random-effects model was applied. Because the units of the scales in the included studies were different, pooled statistics of *SMD* values were chosen for analysis. The results revealed that there was no statistically significant difference between intervention and control groups in the effect on the quality of life in women with PFD (SMD = 0.95, 95% CI (− 0.55, 2.45), *p* > 0.05). The results are provided in Fig. 6.

### Sensitivity analysis and publication bias

**Fig. 7:**
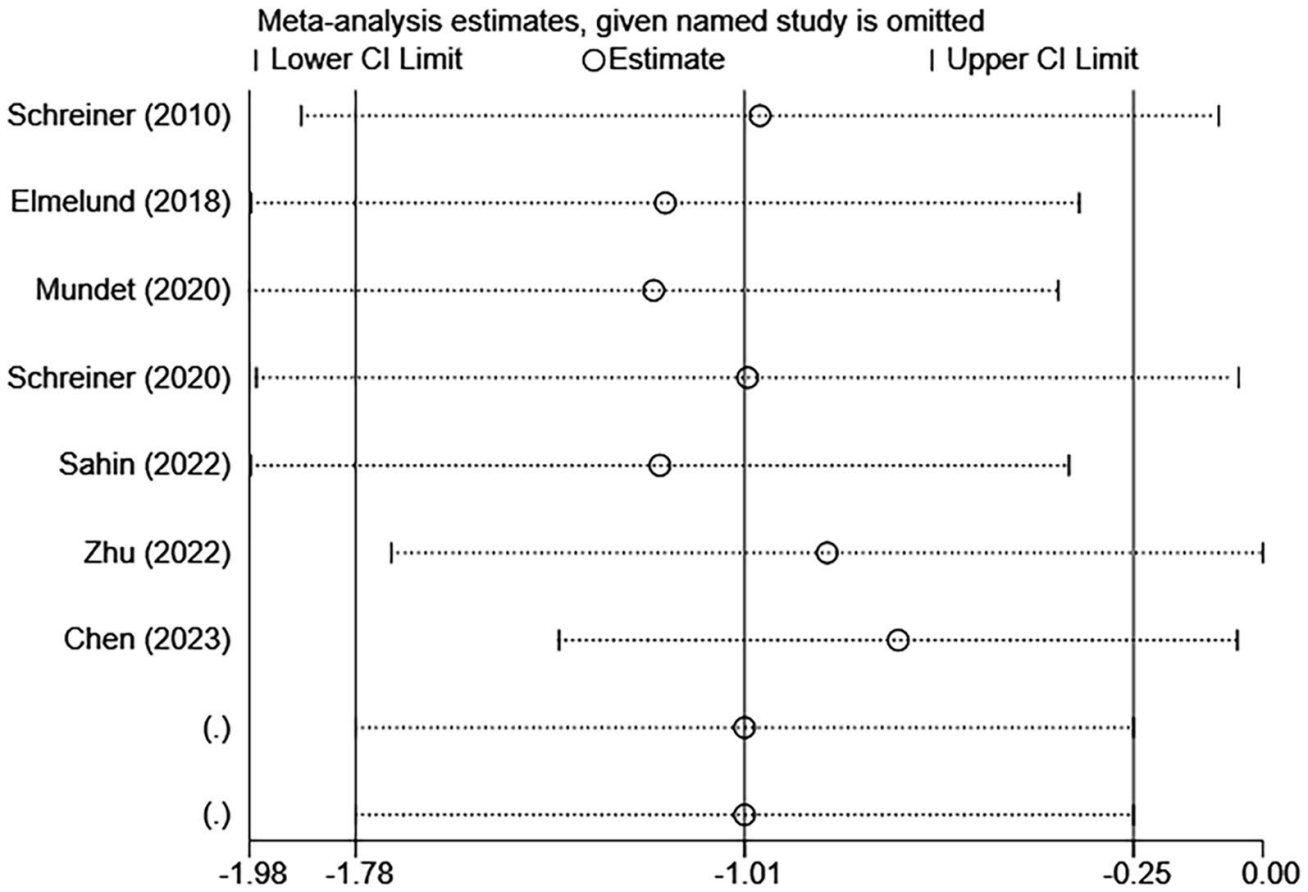
Sensitivity analysis of the PFD severity

**Fig. 8:**
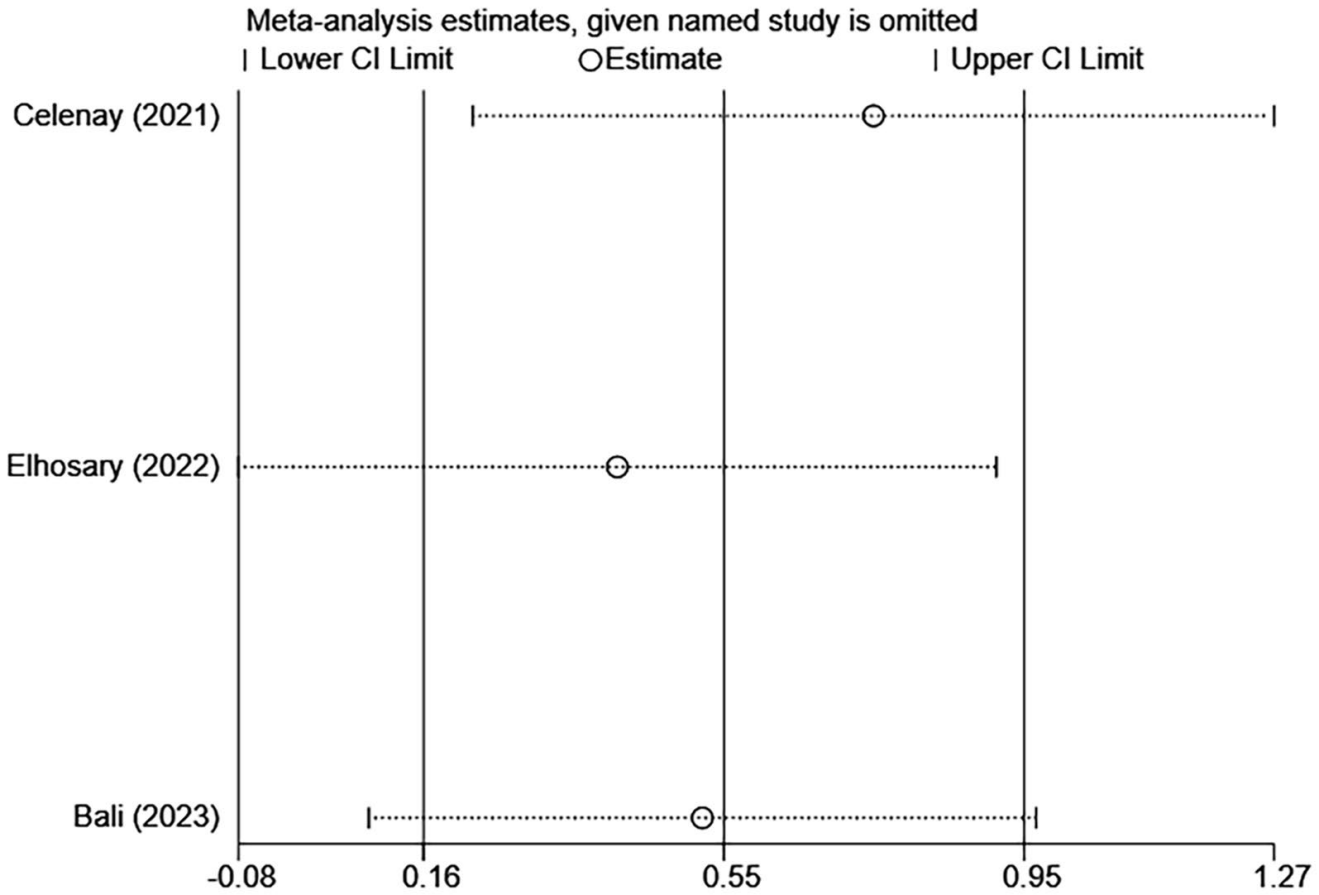
Sensitivity analysis of pelvic floor muscle strength

**Fig. 9:**
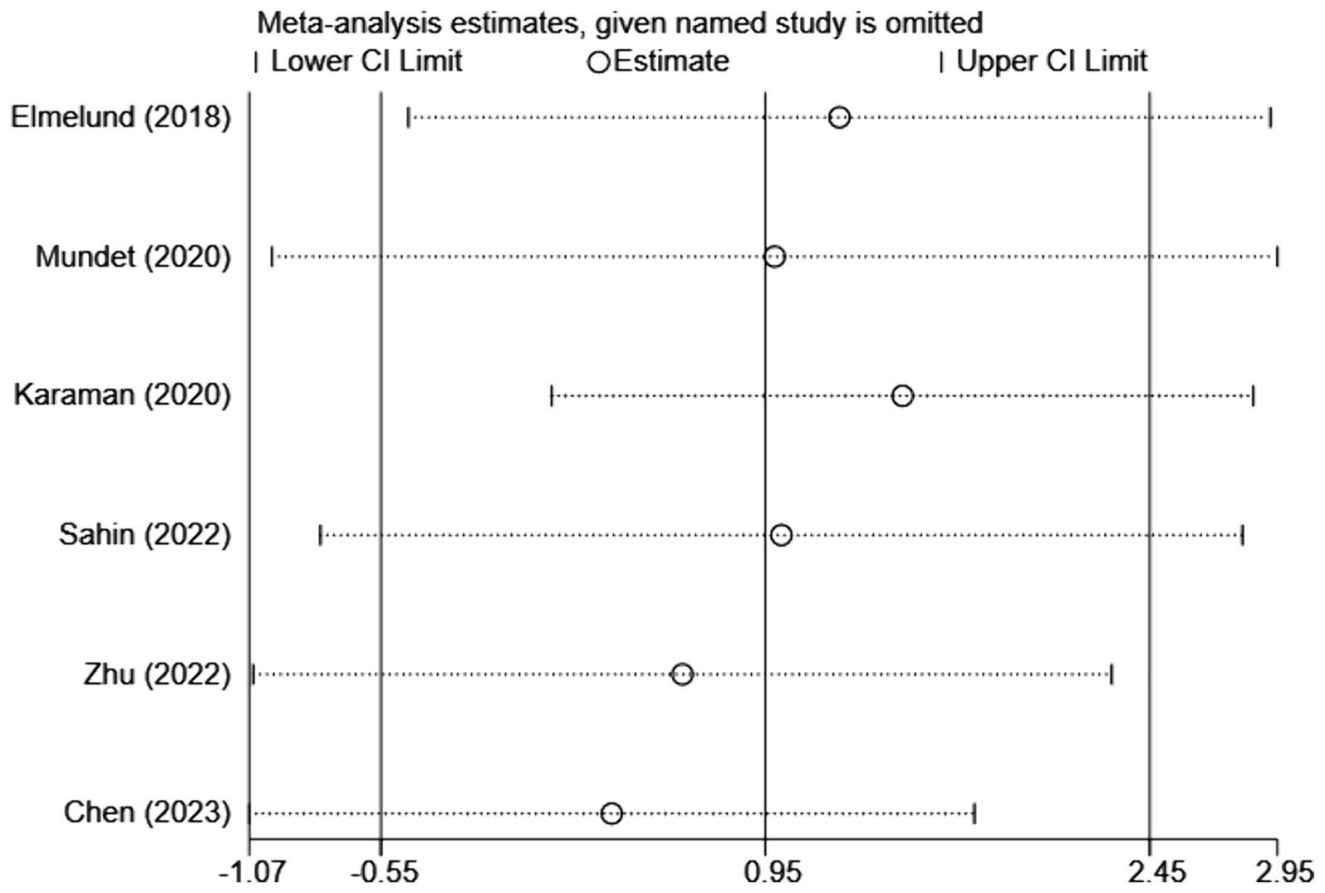
Sensitivity analysis of quality of life

Sensitivity analysis was conducted to assess the potential influence of individual studies on pooled data. It was clear that the results of the PFD severity, pelvic floor muscle strength, and quality of life were stable when the studies were removed one by one (Figs. 7, 8, 9). In this study, Egger’s funnel plot was not used to evaluate publication bias, as fewer than ten studies were included.

## Discussion

Despite many original studies on the effects of ES combined with PFME on the treatment of female PFD, there is currently no meta-analysis available on this treatment approach for these patients. Therefore, this is the first meta-analysis that studies the effect of ES combined with PFME on the treatment of female PFD patients. In this meta-analysis with a systematic review, 12 RCTs from four databases were included and analyzed. The majority of these studies (75%) were published within the past 5 years, which indicated the increase in usage and acceptance of ES combined with PFME as a preferred treatment option of female PFD patients. In this study, compared with PFME alone, ES combined with PFME was found to be significant in reducing the severity of PFD and enhancing pelvic floor muscle strength.

The combination of ES and PFME showed a positive effect in improving the PFD severity and strengthening pelvic floor muscles in female PFD patients (*P* < 0.05) as compared with PFME alone. The reason is that ES, on the one hand, can stimulate the release of acetylcholine in peripheral nerves, promoting the contraction of pelvic floor muscles and restoring the proprioception of Type I and II pelvic floor muscle fibers; on the other hand, it can stimulate or inhibit sympathetic nerve pathways, regulating bladder contraction force and increasing the metabolic level of the detrusor muscle [21, 22]. Furthermore, as a preferred active exercise for PFD patients, PFME can enhance the contraction force of pelvic floor muscles [25]. Therefore, the combination can effectively improve the function of pelvic floor muscles in female patients and increase muscle contraction force. The improvement brought by PFME requires long-term persistence and depends on the patient's initiative. At the same time, related studies showed that over 25% of participants were unable to understand how to contract the pelvic floor muscles under verbal instruction [26]. Therefore, the improvement of pelvic floor muscle function using PFME alone was not significant. Furthermore, we found that there was high heterogeneity when assessing the improvement of the PFD severity in female patients with the combination of ES and PFME, which might be related to the frequency of weekly interventions. When the intervention frequency was once a week [16], there was a statistical difference in improving the PFD severity in female patients between the intervention and control groups (*P* < 0.05); however, when the intervention frequency was five times a week [11], there was no statistical difference between the two groups (*P* > 0.05). This could be a major cause of heterogeneity.

There was no significant difference in improving the quality of life in female PFD patients with ES combined with PFME (*P* > 0.05). The intervention of ES mostly involves stimulating the tissue around the vagina and the pelvic floor at the same time. Doctors need to insert a device with stimulants into the vagina, which increases the patients' physical discomfort and psychological pressure, reducing treatment compliance and thereby impacting their quality of life. In addition, the high heterogeneity identified in the results may be due to the variety of tools used to assess quality of life, which would affect the reliability of the results.

Above all, the clinical usefulness of the combination of ES and PFME for female patients with PFD is mainly reflected in the reduction of PFD symptoms and the enhancement of pelvic floor muscle strength. This is crucial for the prevention of complications such as uterine prolapse and urinary incontinence in the later stages of the disease. In addition, compared with the traditional PFME alone, ES may provide immediate feedback on muscle activity. This is helpful for personalized rehabilitation training and the improvement of the treatment compliance of patients, thereby obtaining better long-term efficacy.

Besides, there were some limitations in this meta-analysis. First, there may be differences in the ES interventions, including different intensities, frequencies, and study populations, which might affect the accuracy of the evidence. Second, the study only included studies in English, which might introduce some bias in the results. Finally, due to the limited number of included studies, it is hoped that more high-quality studies can be included in the future to increase the reliability of the results. Despite these limitations, our study offers valuable insights for clinical practice.

## Conclusion

Compared with PFME, the combination of ES and PFME could effectively reduce the severity of female PFD. This combination treatment has a certain positive effect on improving female patients’ pelvic floor muscle strength. More high-quality studies are needed in the future to further substantiate these findings.

### Supplementary Information

Additional file 1. Table S1. Subject words and free words for retrieval strategy.

## Data Availability

The original contributions presented in the study are included in the article/Supplementary Material. Further inquiries can be directed to the corresponding author.
