# Supplementary material for: Meta-analysis of the therapeutic effect of electrical stimulation combined with pelvic floor muscle exercise on female pelvic floor dysfunction
Source: Eur J Med Res. 2024 Jul 22;29:380. doi: 10.1186/s40001-024-01979-1 (PMC11265018; doi:10.1186/s40001-024-01979-1)
Supplement: Supplementary file 1 — Additional file 1. Table S1. Subject words and free words for retrieval strategy. [file 40001_2024_1979_MOESM1_ESM.docx]

Table S1 Subject Words and Free Words for Retrieval Strategy

| Subject Words | Free Words |
| --- | --- |
| "Exercise Therapy"[Mesh] | Exercise Therapy[Title/Abstract]  Remedial Exercise*[Title/Abstract  Exercise Therap*[Title/Abstract]  Rehabilitation Exercise*[Title/Abstract]  pelvic floor exercise*[Title/Abstract]  PFE[Title/Abstract]  pelvic floor muscle training[Title/Abstract]  pelvic floor muscle exercise*[Title/Abstract]  pelvic floor training[Title/Abstract]  pelvic muscle training[Title/Abstract]  pelvic floor muscle training[Title/Abstract] |
| "Electric Stimulation"[Mesh]  "Electric Stimulation Therapy"[Mesh] | Electr*Stimulation*[Title/Abstract]  electrostimulus[Title/Abstract]  galvanostimulation[Title/Abstract] electrostimulation[Title/Abstract]  Therap* Electric* Stimulation[Title/Abstract]  Electrotherapy[Title/Abstract]  Interferential Current Electrotherapy[Title/Abstract]  electro therapy[Title/Abstract]  electrostimulation therap*[Title/Abstract] |
| "Pelvic Floor Disorders"[Mesh]  "Pelvic Girdle Pain" [MeSH]  "Pelvic Organ Prolapse" [MeSH]  "Pelvic Pain" [MeSH]  "Urinary Incontinence" [MeSH]  "Urinary Incontinence, Stress[MeSH]  "Urinary Incontinence, Urge" [MeSH]  "Fecal Incontinence" [MeSH]  "Prolapse" [MeSH]  "Sexual Dysfunction, Physiological" [MeSH] | Pelvic Floor Disorder*[Title/Abstract]  Pelvic Floor Disease*[Title/Abstract]  pelvic diaphragm d?sfunction[Title/Abstract]  pelvic floor d?sfunction*[Title/Abstract]  pelvic wall disease[Title/Abstract]  Pelvic Girdle Pain*[Title/Abstract]  Symphysis Pubis Dysfunction*[Title/Abstract]  Pelvic Organ Prolapse*[Title/Abstract]  Urogenital Prolapse*[Title/Abstract]  Vaginal Vault Prolapse*[Title/Abstract]  colpoptosis[Title/Abstract]  POP[Title/Abstract]  Pelvi* Pain*[Title/Abstract]  bladder incontinence[Title/Abstract]  incontin* urin*[Title/Abstract]  involuntary urin*[Title/Abstract]  Urinary Stress Incontinence[Title/Abstract]  Urinary Reflex Incontinence[Title/Abstract]  urge urin* incontinence[Title/Abstract]  Bowel Incontinence[Title/Abstract]  anal incontinence[Title/Abstract]  Prolapse*[Title/Abstract]  Physiological Sexual Dysfunction*[Title/Abstract]  Physiological Sexual Disorder*[Title/Abstract]  Sex* Disorders[Title/Abstract]  coital disorder[Title/Abstract] |
